# Engineering microparticles based on solidified stem cell secretome with an augmented pro-angiogenic factor portfolio for therapeutic angiogenesis

**DOI:** 10.1016/j.bioactmat.2022.03.015

**Published:** 2022-04-02

**Authors:** Thomas Später, Marisa Assunção, Kwok Keung Lit, Guidong Gong, Xiaoling Wang, Yi-Yun Chen, Ying Rao, Yucong Li, Chi Him Kendrick Yiu, Matthias W. Laschke, Michael D. Menger, Dan Wang, Rocky S. Tuan, Kay-Hooi Khoo, Michael Raghunath, Junling Guo, Anna Blocki

**Affiliations:** a Institute for Clinical & Experimental Surgery, Saarland University, Homburg, Saar, Germany; b School of Biomedical Sciences, Faculty of Medicine, The Chinese University of Hong Kong, Shatin, New Territories, Hong Kong, China; c Institute for Tissue Engineering and Regenerative Medicine, The Chinese University of Hong Kong, Shatin, New Territories, Hong Kong, China; d BMI Center for Biomass Materials and Nanointerfaces, College of Biomass Science and Engineering, Sichuan University, Chengdu, Sichuan, 610065, China; e Bioproducts Institute, Departments of Chemical and Biological Engineering, The University of British Columbia, Vancouver, BC, Canada; f Academia Sinica Common Mass Spectrometry Facilities for Proteomics and Protein Modification Analysis, and Institute of Biological Chemistry, Academia Sinica, Nankang, Taipei, China; g Shun Hing Institute of Advanced Engineering (SHIAE), Faculty of Engineering, The Chinese University of Hong Kong, Shatin, New Territories, Hong Kong, China; h Ministry of Education Key Laboratory for Regenerative Medicine, School of Biomedical Sciences, The Chinese University of Hong Kong, Shatin, New Territories, Hong Kong, China; i Department of Orthopaedics & Traumatology, The Chinese University of Hong Kong, Shatin, New Territories, Hong Kong, Hong Kong Special Administrative Region of China; j Institute for Chemistry and Biotechnology, Zurich University of Applied Sciences, Wädenswil, Switzerland; k State Key Laboratory of Polymer Materials Engineering, Sichuan University, Chengdu, Sichuan, 610065, China

**Keywords:** Dextran sulfate, Extracellular matrix, Mesenchymal stem cells, Therapeutic angiogenesis, Wound healing, Poly-electrolyte-driven co-assembly

## Abstract

Tissue (re)vascularization strategies face various challenges, as therapeutic cells do not survive long enough *in situ*, while the administration of pro-angiogenic factors is hampered by fast clearance and insufficient ability to emulate complex spatiotemporal signaling. Here, we propose to address these limitations by engineering a functional biomaterial capable of capturing and concentrating the pro-angiogenic activities of mesenchymal stem cells (MSCs).

In particular, dextran sulfate, a high molecular weight sulfated glucose polymer, supplemented to MSC cultures, interacts with MSC-derived extracellular matrix (ECM) components and facilitates their co-assembly and accumulation in the pericellular space. Upon decellularization, the resulting dextran sulfate-ECM hybrid material can be processed into MIcroparticles of SOlidified Secretome (MIPSOS). The insoluble format of MIPSOS protects protein components from degradation, while facilitating their sustained release. Proteomic analysis demonstrates that MIPSOS are highly enriched in pro-angiogenic factors, resulting in an enhanced pro-angiogenic bioactivity when compared to naïve MSC-derived ECM (cECM). Consequently, intravital microscopy of full-thickness skin wounds treated with MIPSOS demonstrates accelerated revascularization and healing, far superior to the therapeutic potential of cECM. Hence, the microparticle-based solidified stem cell secretome provides a promising platform to address major limitations of current therapeutic angiogenesis approaches.

## Highlights

• Dextran sulfate assembles with mesenchymal stem cell secretome.
• As a result, microparticles of solidified stem cell secretome (MIPSOS) are formed.
• The insoluble MIPSOS format protects proteins from premature degradation.
• MIPSOS are enriched in pro-angiogenic factors and exhibit gradual release kinetics.
• MIPSOS demonstrate superior pro-angiogenic properties and thus therapeutic potential.

## Graphical abstract

A high molecular weight sulfated polyglucose polymer, dextran sulfate, co-assembles with mesenchymal stem cell derived secretome into insoluble microparticles (named MIPSOS). Simultaneously, dextran sulfate's ability to mimic heparan sulfate promotes the accumulation of a plethora of pro-angiogenic factors, resulting in superior pro-angiogenic properties and thus therapeutic potential.

**Figure:**
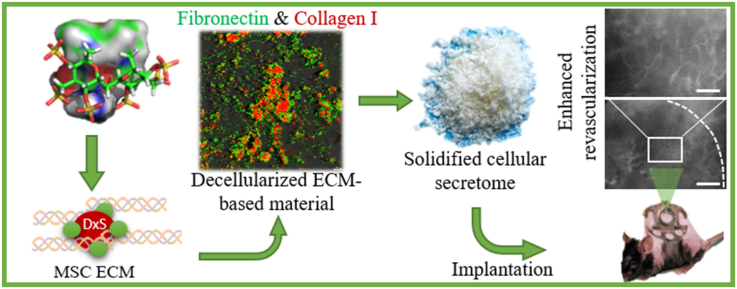

## 1 Introduction

**Fig. 1:**
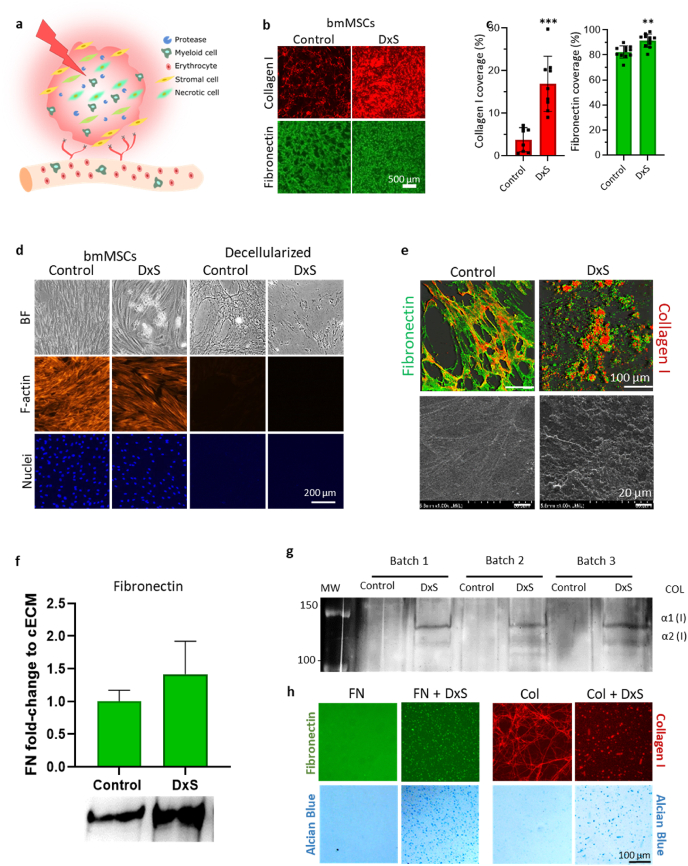
Dextran sulfate-mediated deposition and assembly of MSC-derived ECM. (a) Schematic of an ischemic injury. Impaired vascularization of tissue leads to necrotic cells, which expand from the center into the periphery. This promotes an inflammatory response characterized by myeloid cell infiltration and enhanced proteolytic activity. (b) Immunofluorescence staining of collagen type I (red) and fibronectin (green) deposited by cultured bone marrow-derived mesenchymal stem cells (bmMSC) on day 6 in the absence (control) and presence of dextran sulfate (DxS). Bar = 500 μm (c) Corresponding quantification of stained surface area per field of view. (d) Decellularized ECM. Complete decellularization of assembled ECM is evidenced by the absence of F-actin and nuclear (DAPI) staining. BF, bright-field microscopy. Bar = 200 μm. (e) Collagen type I and fibronectin immunostaining and topography of decellularized ECM. Confocal microscopy (top) and scanning electron microscopy (bottom) of decellularized ECM of control and dextran sulfate-treated bmMSC cultures (DxS). Bar = 100 μm (top) or 20 μm (bottom). (f) Western blot analysis of fibronectin (FN) in decellularized control and dextran sulfate-supplemented cultures (DxS) and densitometric analysis of immune-reactive bands (n = 3). (g) Silver stained SDS-PAGE of pepsin-digested samples of control ECM and ECM deposited in the presence of dextran sulfate (DxS) showing collagen type I (COL) α1 and α2 bands. (h) Purified fibronectin (FN) or collagen type I (Col) were incubated with dextran sulfate (100 μg/ml) and deposited structures were visualized by immunocytochemistry. Deposited dextran sulfate was visualized by staining with Alcian Blue at a pH 2.5 before immunostaining. *, p < 0.05; **, p < 0.01; ***, p < 0.001.

Chronic ischemic conditions, such as coronary artery, cerebrovascular, and peripheral vascular diseases are leading causes of death and disability worldwide [1,2]. As revascularization is dysregulated and insufficient, the resultant malperfusion leads to cell death and growth of necrotic tissue areas, further impairing healing and regeneration [3] (Fig. 1a). Therefore, various revascularization strategies, such as the delivery of pro-angiogenic factors and cell-based therapy, have been explored. Treatments based on the delivery of growth factors have so far shown limited clinical efficacy due to their rapid diffusion and short half-life *in vivo* [4]. In the quest to remedy this, pro-angiogenic factors were delivered via gene-therapy [5,6], incorporated into delivery systems [4,5,[7], [8], [9], [10]], or were genetically engineered to display increased affinity to cell surface proteoglycans [11]. However, strategies based on the delivery of single growth factors (or the combination of a few) do not sufficiently capture the complexity of signals and temporal coordination required to direct intricate biological processes, such as revascularization [4]. In addition, administration of growth factors in supra-physiological doses can bring about serious side-effects [12].

Interestingly, under physiological conditions, signaling factors are stabilized and their signaling modulated by being associated with the extracellular matrix (ECM). Indeed, the accurate organization of the ECM is a prerequisite to harness the full complex bioactivity of a wide plethora of factors, while none of them is presented in supra-physiological concentrations, and ensure their long-term stability [13,14]. Initial studies on biomaterials based on tissue-derived ECM have demonstrated promising results in the treatment of ischemic diseases [[15], [16], [17], [18]]. Unfortunately, the use of tissue-derived ECM has inherent challenges, including risk of disease transmission and immune-rejection. Tissue-derived ECM is also set in its composition and cannot be customized toward a specific bioactivity and application [19].

The delivery of stem cells originally promised a more holistic approach, whereby cells may locally secrete a wide range of paracrine factors at physiologically relevant levels [20]. Adult mesenchymal stem cells (MSCs) are of particular interest because of their strongly pro-angiogenic secretome [21,22]. However, MSC-based therapies have remained challenging, as the hostile ischemic microenvironment also compromises cellular survival upon implantation [20]. Furthermore, autologous MSC-based therapies require a preparation time of at least 4–8 weeks, which can be prohibitively long to meet a therapeutic window [23,24]. Moreover, patients requiring cell-based therapy often present with co-morbidities that can additionally impair autologous cell functionality. Therefore, allogenic cell therapy would represent an attractive scalable approach with predictable functionalities. However, potential disease transmission and immune rejection pose increased regulatory hurdles [23,24]. Against this background, strong interest has arisen to engineer functional materials, which can utilize and capture the pro-angiogenic activities of MSCs in a rapid and scalable manner that does not pose safety concerns generally associated with cell-based therapy or the utilization of soluble signaling factors and tissue-derived ECM [20].

To this end, it is important to note that MSCs are active extracellular matrix (ECM) producers *in vitro*, and that MSC-produced ECM at least partially retains the beneficial intrinsic properties of the native ECM [[25], [26], [27]], including storage and stabilization of signaling factors. A MSC-derived ECM-based biomaterial has thus the potential to exhibit the beneficial pro-regenerative properties of the complex MSC secretome, while the intrinsic insolubility of ECM will protect bioactive factors from pre-mature degradation and enable their sustained release. Furthermore, the proper assembly of all components will allow full exploitation of their bioactivity, with none of the factors being present in supra-physiological doses [25,27].

Herein, we introduced the sulfated polyglucose polymer, dextran sulfate (500 kDa) into MSC cultures to aggregate and co-precipitate major ECM components into the pericellular space. This polyelectrolyte-mediated co-assembly resulted in amplified amounts of MSC-derived ECM *in vitro* [28], which could be further processed into microparticles of solidified secretome (MIPSOS). We hypothesized that concurrently with the dextran sulfate-ECM co-assembly, dextran sulfate's ability to mimic heparan sulfate [[29], [30], [31], [32], [33], [34]] will facilitate the accumulation of various pro-angiogenic factors within MIPSOS, resulting in an enhanced pro-angiogenic bioactivity and thus a superior pro-healing and regenerative potential. Indeed, comparison of the proteomes of MIPSOS and naïve ECM assembled in the absence of dextran sulfate (cECM) confirmed the accumulation of pro-angiogenic factors within MIPSOS. Some of the identified factors were known members of the pro-regenerative secretome of MSCs, while others were hitherto not implicated with MSCs. The insoluble format of MIPSOS protected the integrated secretome components from premature degradation and enabled their controlled release when encapsulated in a hydrogel. Intravital fluorescence imaging of perfused blood vessels during skin wound healing in mice implanted with MIPSOS demonstrated accelerated and enhanced revascularization as compared to cECM, resulting in augmented wound healing. Our findings demonstrate the feasibility of an innovative and straightforward approach to engineer solidified stem cell secretome enriched in pro-angiogenic factors, thus addressing major limitations of stem cell, soluble signaling factor and tissue-derived ECM-based revascularization strategies.

## 2 Materials and methods

*Cell culture and ECM-based particle synthesis.* Human bone marrow MSCs from 3 donors (Lonza, Walkersville, USA; and Millipore, Temecula, USA) were expanded individually in supplemented Dulbecco's Modified Eagle's medium (DMEM, GlutaMAX, 1 g/L glucose, 10% FBS, penicillin/streptomycin, all reagents from Gibco, Grand Island, USA), at 37 °C, 5% CO_2_. At passage 7 cells were seeded at 7000 cells/cm^2^. 24 h later, media was switched to ECM induction medium (DMEM, 0.5% FBS, 0.1 mM ascorbic acid; cat. #A8960, Sigma-Aldrich, St. Louis, USA), with or without dextran sulfate (10 μg/ml, 500 kDa, Sigma-Aldrich, cat. #D8906S, Steinheim, Germany). After 6 days, cultures were decellularized using 0.1% (w/v) deoxycholate in deionized water (cat. #30970, Sigma-Aldrich, Germany), supplemented with protease inhibitor cocktail (cat. #P8340, Sigma-Aldrich, USA), by incubating cells for 15–20 min at room temperature until cell removal was clearly visible under the microscope. Remaining nucleic material was removed by incubating decellularized matrices with 0.04 mg/ml DNAse I (cat. #LS002007, Worthington, Lakewood, USA) dissolved in PBS with Ca^2+^/Mg^2+^ for 15 min at 37 °C. Decellularized matrices were then washed once with PBS followed by one wash with double deionized water (ddH_2_O), and mechanically harvested in ddH_2_O by using a cell scraper. This resulted in the breakdown of the decellularized matrices into microparticles, which were then collected, lyophilized and stored at −20 °C.

*Live Dead Assay.* Cell viability of MSCs in the presence and absence of dextran sulfate were assessed using LIVE/DEAD™ Viability/Cytotoxicity Kit, for mammalian cells (cat. #L3224, Invitrogen, Life technologies, USA), according to the manufacturer's protocol. Images were taken by epifluorescence microscopy (Olympus IX83; Olympus, Tokyo, Japan).

*Cytochemistry.* Intact or decellularized cell cultures were fixed in 4% paraformaldehyde for F-actin staining or with ice-cold methanol for immunocytochemistry. For the latter, blocking in 3% bovine serum albumin (BSA) for 1 h was followed by 18 h incubation with primary antibodies (Table 1; 4 °C, in 1% BSA), PBS washes and subsequent incubation with secondary antibodies (Table 1; 2 h) and PBS washes. F-actin was directly stained with fluorescently labeled phalloidin (Table 1) for 30 min, followed by PBS washes. Samples were stored and examined in PBS by epifluorescence microscopy (Olympus IX83; Olympus, Tokyo, Japan) or using a confocal microscope (Leica TCS SP8; Leica Microsystems, Wetzlar, Germany).

**Table 1:** Antibodies used. IF – immunofluorescence; WB – Western blot; Suppliers: Abcam, Hong Kong; Molecular Probes, Eugene, OR, USA; BD Pharmingen, San Diego, CA, USA; Sigma-Aldrich, St. Louis, MO, USA; Dianova, Hamburg, Germany;^†^ Life Technologies, Ober-Olm, Germany.

Reagents	Host	Dilution used	Catalog #	Supplier
Primary antibodies				
anti-fibronectin	rabbit	1:500 (IF)	ab2413	Abcam
		1:6000 (WB)		
anti-Collagen I	mouse	1:700	C2456	Sigma
anti-mouse CD31	rat	1:100	DIA-310	Dianova
**Secondary antibodies**				
anti-rabbit AF 488		1:500	ab150077	Abcam
anti-mouse AF 647		1:1000	A31571	Molecular Probes
anti-rabbit-HRP		1:5000	A27036	Molecular Probes
anti-rat IgG Alexa 555		1:30	A-21434	Life Technologies^†^
Hoechst33342 (cell nuclei)		2 μg/ml	875756-97-1	Sigma-Aldrich
**Others**				
Phalloidin-AF 555		1:1000	ab176756	Abcam
DAPI		1:1000	564907	BD Pharmingen

*Sodium dodecyl sulfate–polyacrylamide gel electrophoresis (SDS-PAGE) and silver staining.* Decellularized cultures were digested with 0.25 mg/ml pepsin (Promega, cat. #V195A, Madison, USA, in 0.25 N HCl, 0.5% TritonX-100) for 3 h at room temperature. After neutralization with NaOH, equal volumes of peptic extracts were resolved by SDS-PAGE (instruments and reagents by Life Technologies, Rockford, USA). Briefly, samples were boiled in Laemmli sample buffer containing 10% 2-mercaptoethanol for 5 min and resolved in an 8% polyacrylamide gel. Protein bands were visualized using Silver Staining Plus kit (cat. #161–0449, Bio-Rad, USA).

*Western blotting.* Cell culture extracts were diluted in Laemmli sample buffer with protease inhibitor cocktail and 10% 2-mercaptoethanol. Upon determining protein concentration by a Pierce™ Rapid Gold BCA Kit (Life Technologies, USA) equal amounts of protein were loaded and resolved by SDS-PAGE as described above. After transfer to a PVDF membrane in a Power Blotter (Life Technologies, USA), proteins were detected immunochemically. Stained bands were visualized with ECL Super Signal West Pico Plus (cat. #34580 Life Technologies, USA) and densitometrically quantified using Image Lab Software v6.0.1 (Bio-Rad, USA).

*Scanning electron microscopy.* Decellularized cultures on plastic coverslips were air dried, sputter-coated with gold and visualized in a Hitachi SU8010 Cold Field Scanning Electron Microscope (Hitachi, Japan).

*Cell-free dextran sulfate experiments.* Dxtran sulfate-fibronectin and dextran sulfate-collagen I interactions in a cell-free system were performed by incubating tissue culture treated plates with DMEM supplemented with 30 μg/ml of fibronectin from bovine plasma (Saint Louis, MO 63103, USA) or 30 μg/ml telocollagen I (San Diego, CA92150, Cat. No. 5225) with 100 μg/ml dextran sulfate for 16 h at 37 °C. The plates were then washed with PBS and fixed with 4% paraformaldehyde in PBS for 15 min at room temperature. Samples were stained with Alcian Blue (1% in 3% actic acid, pH 2.5) followed by immunostaining as described above.

*Transmission electron microscopy.* Collagen I (cat. #C8062, Beijing Solaibao Technology Co., Beijing, China) was dissolved to 0.2 mg/ml in 6.0 mM acetic acid with or without dextran sulfate (10 μg/ml), neutralized (pH 7.4) and incubated at 37 °C for 24 h. 10 μl of collagen I solution were dropped on formvar-carbon-coated copper grids (50 mesh, cat. #AGH50, Beijing Zhongjingkeyi Technology Co., Ltd., China) and then stained with a phosphotungstic acid solution (1%, 10 μl) for 3 min. After that, the residual phosphotungstic acid solution was removed with filter papers, and samples were dried under an infrared lamp (about 1 min). The images were acquired in FEI Tecnai G2 F20 S-TWIN (USA).

*UV and circular dichroism spectroscopy (CD).* Collagen I was dissolved in acetic acid as described, with or without dextran sulfate (10 μg/ml) and incubated at 37 °C for 24 h. UV and CD spectra were obtained from circular dichroism spectrophotometer J-815 (JASOC, Japan).

*Molecular dynamics (MD) simulation.* The simulation was performed using Gromacs 2018.4 software, Amber99SB-ILDN force field; TIP3P model was used for water molecules. Firstly, the system was placed in the center of a cube with 7 nm margins, which was randomly filled with water molecules. The water molecules were replaced with suitable ions to make the simulated system electrically neutral. Then, the system was simulated and optimized by the method of energy minimization with the steepest descent method, so that the whole system was in the state of the lowest energy. The system was equalized under canonical, isothermal and isobaric ensemble using restricted MD simulation. Verlet frog leaping algorithm was used to solve Newton's equations of motion, and the integral step was set as 2 fs. Lennard-Jones function was used to calculate van der Waals forces, and the non-bond truncation distance was set as 1.2 nm. The Lincs algorithm was used to constrain the bond lengths of all atoms, and the Particle Mesh Ewald method was used to calculate long-range electrostatic interactions. The lattice width was set as 0.16 nm. Periodic boundary conditions were used in the simulation process. All MD simulations were performed in an isothermal and isobaric ensemble at 300 K and 1 atm under the control of temperature and pressure by the V-Resale and Parrinello-Rahman methods, respectively, with coupling constants of 0.1 and 0.5 ps. The simulation time of molecular dynamics was 100 ns.

*Urea-extraction.* ECM remaining in the flask after decellularization was washed with ddH_2_0 and overlaid with 4 M urea (0.1 ml/cm^2^, cat. #IB72060, IBI, USA) at 4 °C for 3 days. The supernatant was collected and stored at −80 °C. The remaining ECM was mechanically collected into 8 M urea (0.5 ml/cm^2^), filled into tubes and extracted for 3 days at 4 °C under agitation. Non-solubilized components were spun down and the urea solutions containing extracts were combined and dialyzed against ddH_2_0 (3500 KDa cut-off dialysis tubing, 3 days) and subsequently lyophilized and stored at −20 °C.

*Concentration of conditioned media.* Conditioned media from a 6-day culture of bmMSC was collected, lyophilized and resuspended in ddH_2_0 at 10% of its initial volume. Concentrated conditioned media (CCM) was dialyzed as above to remove inorganic components until the medium turned colorless. The dialysate was again lyophilized and stored at −20 °C.

*Degradation assay.* ECM-based microparticles, ECM urea-extracts, and CCM were suspended in PBS at 10 mg/ml. The suspension was incubated in a thermoshaker (Thermofisher Scientific, USA) at 37 °C and agitated at 400 rpm. Samples were collected on day 0 (before incubation), 1, 2 and 3 and stored immediately at −20 °C until further use. Next, samples were diluted in lithium dodecyl sulfate sample buffer (Life Technologies, USA) with 10% MSH, heat denatured and separated in 4–12% gradient PAGE (NuPAGE, Life Technologies, USA) in 2-(*N*-morpholino)ethanesulfonic acid (MES) running buffer at 200 V. Protein bands were detected by silver staining as described.

*Protein release assay.* ECM-based microparticles, ECM urea-extracts, and CCM were suspended in PBS at 5 mg/ml. Collagen I solution was prepared as 80% stock solution, 10% gel neutralization solution (cat. #5225, Advanced BioMatrix, CA, USA) and 10% of 10xPBS. ECM material or CCM solution were mixed with collagen I solution and added into 96-well plate wells at 50 μl/well. After polymerization at 37 °C for 40 min, collagen hydrogels were overlaid with 50 μl PBS. 30 μL of the supernatant was collected at various time-points and replenished with fresh PBS. Proteins released into the supernatant from ECM material or CCM were quantified with the Pierce™ Rapid Gold BCA Kit (Life Technologies, USA). As the BCA assay almost exclusively captures amino acids that are extremely rare in collagen [35], the assay is not confounded by the presence of a collagen hydrogel. However, values from empty collagen hydrogels served for normalization, and the total amount of released protein was calculated.

*Endothelial cell proliferation.* Human umbilical vein endothelial cells (HUVECs; cat. #PCS-100-013, ATCC, Virginia, USA) below passage 8 were seeded onto the ECMs at 7000 cells/cm^2^ and cultured for 2 days at 37 °C, 5% CO_2_. After 2 days, medium was replaced with Cell Counting Kit 8 (cat. #K1018, ApexBio, Houston, USA) in endothelial growth medium 2 (EGM2, cat. #CC-3162, Lonza, USA) and incubated for additional 2 h at 37 °C, 5% CO_2_. Resulting medium was then transferred to 96-well plates for absorbance measurement at 450 nm.

*Endothelial cell spheroid sprouting assay.* HUVECs were expanded in EGM2 and seeded at passages 5–8 into spheroid plates (Sphericalplate 5D, Kugelmeiers, Zürich, Switzerland) at 700 cells/micro-well. HUVEC spheroids were collected after 24 h, embedded in a 1 mg/ml collagen I gel (cat. #5225, Advanced BioMatrix, CA, USA) containing EGM2 at 20 spheroids/250 μL and finally cast onto decellularized ECMs in a 24-well plate well. Cell-free polystyrene well bottoms served as controls. The hydrogel polymerized for 2 h at 37 °C and then was overlaid with 250 μL of EGM2. Spheroids were allowed to sprout for 24 h. After F-actin staining, the spheroids were imaged using an epifluorescence microscope (Zeiss AxioImager 7). The sprout length was determined using Image J software v1.52i (https://imagej.nih.gov/ij/).

*Differentiation of macrophages from THP-1.* Macrophages differentiation was induced from THP-1 cells using 100 ng/ml phorbol 12-myristate 13-acetate (PMA, cat. # 74042, Stemcell Technologies, Vancouver, Canada) in fresh supplemented RPMI medium. Cells were allowed to differentiate for 24 h at 37 °C, 5% CO_2_. Resulting macrophages were then trypsinized and reseeded at 25,000 cells/cm^2^ and cultured for a further 6 days at 37 °C, 5% CO_2_.

*Gene expression analysis.* Total RNA was extracted from the macrophages using RNAiso Plus (cat. #9108/9109, Takara Bio Inc, Kusatsu, Shiga, Japan), and complementary DNA (cDNA) synthesized with PrimeScript™ RT Master Mix (cat. # RR036A, Takara Bio Inc, Kusatsu, Shiga, Japan) as described by the manufacturer. 50 ng of cDNA and primers listed in Table 2 were used for RT-qPCR with ChamQ SYBR Color qPCR Master Mix (cat. #Q411-03, Vazyme, Nanjing, China) according to the manufacturer's instructions in QuantStudio™ 7 Pro Real-Time PCR System (Applied Biosystems™, Thermofisher, USA). Relative gene expression was calculated using the ΔΔCq method with Design and Analysis Software Version 2.6, QuantStudio 6/7 Pro systems (Applied Biosystem by Thermo Fisher Scientific).

**Table 2:** Primer sequences used in RT-qPCR.

Gene Symbol	Forward Primer	Reverse Primer
IL-10	5′-GACAGACTTGCAAAAGAAGGC-3′	5′-TCTCGAAGCATGTTAGGCAG-3′
TNFa	5′-CAGCCTCTTCTCCTTCCTGA-3′	5′-AGATGATCTGACTGCCTGGG-3′
IL-1b	5′-GAGCTCGCCAGTGAAATGAT-3′	5′-GGAGATTCGTAGCTGGATGC-3′
VEGFA	5′-AGGAGGAGGGCAGAATCATCA-3′	5′-CTCGATTGGATGGCAGTAGCT-3′
FGF2	5′-GTGTGTGCTAACCGTTACCT-3′	5′-GCTCTTAGCAGACATTGGAAG-3′
GAPDH	5-'CCAGGGCTGCTTTTAACTCTGGTAAAGTGG-3′	5′-ATTTCCATTGATGACAAGCTTCCCGTTCTC-3′

*Murine dorsal skinfold chamber implantation.* All animal experiments were approved by the local government (permit number: 36/2019) and conducted in accordance with the European legislation on the protection of animals (Directive 2010/63/EU) and NIH guidelines on the care and use of laboratory animals (NIH publication #85-23 Rev. 1985). Dorsal skinfold chambers were implanted in wild-type C57BL/6 N mice (7 animals per group) (Institute for Clinical and Experimental Surgery, Saarland University, Homburg/Saar, Germany), aged 4–6 months and with a body weight of 24–28 g. The animals were housed under a 12 h light/dark cycle and received water and food *ad libitum* (Altromin, Lage, Germany). For the implantation of the dorsal skinfold chamber (Sorg et al. [36,37]), mice were anesthetized by intraperitoneal injection of ketamine (100 mg/kg body weight; Ursotamin®; Serumwerke Bernburg, Bernburg, Germany) and xylazine (12 mg/kg body weight; Rompun®; Bayer, Leverkusen, Germany). Then the two symmetrical titanium frames (Irola Industriekomponenten GmbH & Co. KG, Schonach, Germany) were fixed on the extended dorsal skinfold, as described previously [38] (Fig. 4a). After a 48-h recovery period, the mice were anesthetized again and a 3 mm full-thickness skin defect was created within the observation window of the chamber using a dermal biopsy punch (kaiEurope GmbH). Subsequently, the defect was filled with 8 μl of collagen I gel (1.8 mg/ml), either empty, or containing cECM and MIPSOS (at 10 mg/ml hydrogels) and allowed to polymerize before the observation window of the chamber was sealed with a removable cover glass. Each wound thus received optionally 80 μg microparticles.

*Stereomicroscopy.* Anesthetized mice were positioned on a Plexiglas stage and the dorsal skinfold chamber was positioned under a stereomicroscope (Leica, M651, Wetzlar, Germany) on day 0 (day of implantation) as well as on days 4, 8 and 12. The chamber tissue was visualized in epi-illumination to identify epithelialized and non-epithelialized areas. Microscopic images were quantitatively analyzed by means of the computer-assisted offline analysis system CapImage (Zeintl, Heidelberg, Germany). The epithelialized area (%) was calculated by the equation: (total implant area - non-epithelialized implant area)/total implant area x 100%.

*Intravital fluorescence microscopy.* After stereomicroscopy, 0.1 ml of fluorescein isothiocyanate (FITC)-labeled dextran (5%; 150,000 Da; Sigma-Aldrich, Taufkirchen, Germany) was retrobulbarily injected into the venous plexus of the anesthetized animals for contrast enhancement by staining of plasma. The microvasculature within the chamber observation window was imaged with a Zeiss Axiotech fluorescent microscope (Zeiss, Oberkochen, Germany) and a charge-coupled device video camera (FK6990; Pieper, Schwerte, Germany) [38]. Image analysis was performed using CapImage (Zeintl). Implant vascularization was assessed in 8 regions of interest (ROIs). ROIs exhibiting red blood cell (RBC)-containing microvessels were defined and counted as perfused ROIs (given in % of all ROIs). The functional microvessel density was determined as the total length of all RBC-perfused microvessels per ROI (given in cm/cm^2^). The diameter (d, in μm) and centerline RBC velocity (v, given in μm/s) of 40 randomly selected microvessels were used to calculate the wall shear rate (y, in s^−1^) by means of the Newtonian definition y = 8 × v/d.

*Histology and immunohistochemistry.* Three μm-thick paraffin sections of formalin-fixed tissue were stained with hematoxylin & eosin (HE) and Sirius red. Sirius red-stained sections were viewed under polarized light to visualize mature collagen fibers. The collagen signal was assessed in relation to normal skin within 4 randomly selected ROIs for each sample using a BX60 microscope (Olympus, Hamburg, Germany) and the imaging software cellSens Dimensions 1.11 (Olympus). Additional sections were immunostained for the endothelial cell marker CD31. The density of CD31^+^ microvessels (given in mm^−2^) was quantified from 6 randomly selected ROIs of each implant type.

*Preparation of decellularized ECM for proteomics.* ECM-based particles were suspended in 8 M urea in 50 mM ammonium bicarbonate (ABC buffer), pH 8.0. Partially solubilized samples were centrifuged at 16,000×*g* (10 min, 4 °C), and the supernatant was transferred into fresh microcentrifuge tubes. This fraction will be referred to as the urea-soluble fraction. Protein concentration of the urea-soluble fraction was determined via Bradford assay. Urea-insoluble pellets were resuspended in 2 M urea and treated with 10 mM dithioerythritol for 1 h at 37 °C to reduce disulfide bonds. Free cysteines were alkylated by adding iodoacetamide at 25 mM and incubating them for 1 h in the dark. After diluting to 2 M urea with ABC buffer, samples were deglycosylated with 3 units of PNGase F (Roche, Germany) at 37 °C for 2 h. Samples were pre-digested with Lys-C (Wako, Japan), incubated at 37 °C for 2 h and then digested with trypsin (Promega, USA) overnight at 37 °C. The pH was adjusted by trifluoroacetic acid (TFA) to 2–3 before peptides were separated using the high pH reversed-phase peptide fractionation kit (Thermo Fisher Scientific, USA). Peptides were loaded to the pre-conditioned spin column. After samples were washed twice with water by low-speed centrifugation (3000×*g*, 2 min), a step gradient of increasing acetonitrile concentrations in triethylamine (5%, 7.5%, 10%, 12.5%, 15%, 17.5%, 20% and 50%) was applied to the column to elute bound peptides into eight different fractions by centrifugation (3000×*g*, 2 min). Each fraction was dried by vacuum centrifugation and desalted with C_18_ Spin Tips (Pierce, USA). Desalted peptides were dried and stored at −20 °C.

*LC-MS/MS analysis.* NanoLC-nanoESi-MS/MS analysis was performed on a Thermo UltiMate 3000 RSLCnano system connected to a Thermo Orbitrap Fusion mass spectrometer (Thermo Fisher Scientific, Bremen, Germany) equipped with a nanospray interface (New Objective, Woburn, MA, USA). Peptide mixtures were loaded onto a 75 μm ID, 25 cm length PepMap C18 column (Thermo Fisher Scientific, USA) packed with 2 μm particles with a pore width of 100 Å and separated using a segmented gradient within 120 min from 5% to 35% solvent B (0.1% formic acid in acetonitrile) at a flow rate of 300 nl/min. The mass spectrometer was operated in the data-dependent mode. Briefly, survey scans of peptide precursors from 350 to 1600 *m/z* were performed at a 240 K resolution with a 2 × 10^5^ ion count target. Tandem MS was performed by isolation window at 1.6 Da with the quadrupole, HCD fragmentation with normalized collision energy of 30, and rapid scan MS analysis in the ion trap. The MS^2^ ion count target was set to 10^4^. Only charge states from 2+ to 6+ were chosen for tandem MS/MS.

*Protein identification and quantification.* Raw mass spectral data files (.raw) were searched using Proteome Discoverer software (version 2.4; Thermo Fisher Scientific, USA) and Sequest-HT using the UniProt *Homo sapiens* database (release 2019_06, 20,368 sequences). Sequest-HT search parameters were: 10 ppm mass tolerance for precursor ions; 0.6 Da for fragmentation mass tolerance; two missed cleavages of trypsin; fixed modification was carbamidomethylation of cysteine; and variable modifications were oxidized methionine, deamidation of asparagine and glutamine, and hydroxylation of lysine and proline. The identification data were filtered to a 1% FDR using Percolator algorithm in Proteome Discoverer software platform. Both unique and razor peptides were selected for protein quantification. Other unmentioned parameters were the Proteome Discoverer default settings.

*Proteome down-stream analysis.* LC-MS/MS output was exported to Excel and proteins were identified using Uniprot (https://www.uniprot.org/) and Matrisome project database (http://matrisome.org/) with the unique protein accession number for their subcellular locations. Secreted proteins were separately shortlisted. Proteins consistently present in all 3 biologically independent replicates were selected for further analyses. From the short-listed proteins, those showing an abundance larger than 3-fold between MIPSOS and cECM were subjected to the PANTHER classification system (v. 16.0 http://pantherdb.org) and the Matrisome Project (In silico Matrisome http://matrisomeproject.mit.edu) under Human Matrisome category. ‘The Matrisome Project’ distinguishes putative ECM proteins into core matrisome proteins (collagen, proteoglycans, and glycoproteins) and matrisome-associated proteins (secreted factors, ECM affiliated proteins, and ECM regulators). Gene Ontology (GO) enrichment analysis was performed (http://geneontology.org) with a false discovery rate (FDR) < 0.05. Pathway analysis was performed using the Kyoto Encyclopedia of Genes and Genomes (KEGG) database in DAVID Bioinformatics software (Resources 6.8, https://david.ncifcrf.gov) with FDR <0.05. The pathway map was generated by program R (v3.6.1, www.r-project.org). Protein-protein interaction analysis (PPI) and protein association network formation were performed by the Search Tool for Retrieval of Interacting Genes/Proteins (STRING v. 11.0; https://string-db.org) with the confidence score threshold set at 0.7 (high). In STRING networks, proteins were represented by nodes and the protein clusters were generated based on the STRING local network cluster tool. Next, an extensive literature review on the angiogenesis-related functions of all short-listed proteins was performed (keywords e.g. “Angiogenesis”, “Revascularization”) in google and google scholar search engines, and PubMed and PubMed Central® archives (National Center for Biotechnology Information Library).

*Statistical analysis.* Statistical analysis was performed with One- or Two-way Analysis of Variance algorithm with confirmed normality and equal variance. Summarized results from at least three independent *in vitro* biological runs are presented, 3 replicates each were compared using Post hoc Tukey tests. Data from 7 animals per group are presented for *in vivo* experiments. *P*-values <0.05 were considered statistically significant. Analyses were performed using GraphPad Prism v8.0 (GraphPad Software, San Diego, CA, USA, www.graphpad.com).

## 3 Results

### 3.1 Formation of a fibrous matrix and resulting MIPSOS from cell-derived ECM

Although MSCs are active ECM producers, *in vitro* cellular assembly of ECM is inefficient [25,39,40]. Previous approaches addressing this limitation introduced macromolecular crowding into cell culture, whereby carbohydrate-based macromolecules exclude volume, resulting in an increase of the local effective concentration of cell-secreted components [[39], [40], [41], [42], [43], [44], [45]]. This has profound effects on molecular interactions and reaction kinetics, resulting in the facilitation of ECM deposition *in vitro* [42,44,46]. Dextran sulfate was originally proposed to also act as a macromolecular crowder [39,[47], [48], [49]]. However, we have recently shown that the ECM deposition-enhancing effects of dextran sulfate might rather be based on its direct electrostatic interaction with various proteins in cell culture, thus promoting the deposition of major ECM components via aggregation and co-precipitation [28,50]. In this work, we explored dextran sulfate's ability to promote the assembly of an insoluble pericellular matrix around MSCs with enhanced bioactivity. For this, MSCs were cultured for 6 days under low serum conditions with optional supplementation of dextran sulfate. This had no adverse effects on cell viability (Suppl. Fig. S1). Immunocytochemistry showed that supplementation of dextran sulfate at a previously optimized concentration of 10 μg/ml (fractional volume occupancy (FVO) of <1%) [28] resulted in an accumulation of major structural components such as collagen type I and fibronectin in the ECM (Fig. 1b and c). A thin collagen type I meshwork was observed in untreated cells, while cultures supplemented with dextran sulfate exhibited the typical granular ECM deposition pattern [28] (Fig. 1b). After successful decellularization, as evident by the removal of F-actin and cell nuclei, these observed ECM structures were retained (Fig. 1d). Indeed, confocal and scanning electron microscopy of decellularized ECM materials confirmed the formation of insoluble multi-protein coarse structures in the presence of dextran sulfate, whereas a thin layer of fibrillar structures was seen in untreated ECM (Fig. 1d and e). Western blot analysis revealed that, when same protein amounts were loaded, decellularized dextran sulfate-ECM hybrid materials retained about 40% more fibronectin than untreated ECM, while pepsin-resistant collagen type I was only detectable in ECM deposited in the presence of dextran sulfate (Fig. 1f and g). Hence, dextran sulfate enriched major structural ECM components.

We have shown previously that incubation of dextran sulfate with purified ECM proteins in a cell-free system resulted in their aggregation [28]. This was also evident in the formation and deposition of granular structures exhibiting an overlapping staining for dextran sulfate, which was supplemented at a 10-fold higher concentration (100 μg/ml) to enable its good visualization by Alcian Blue, with fibronectin or collagen type I (Fig. 1h).

### 3.2 Intermolecular interaction of collagen type I and dextran sulfate

**Fig. 2:**
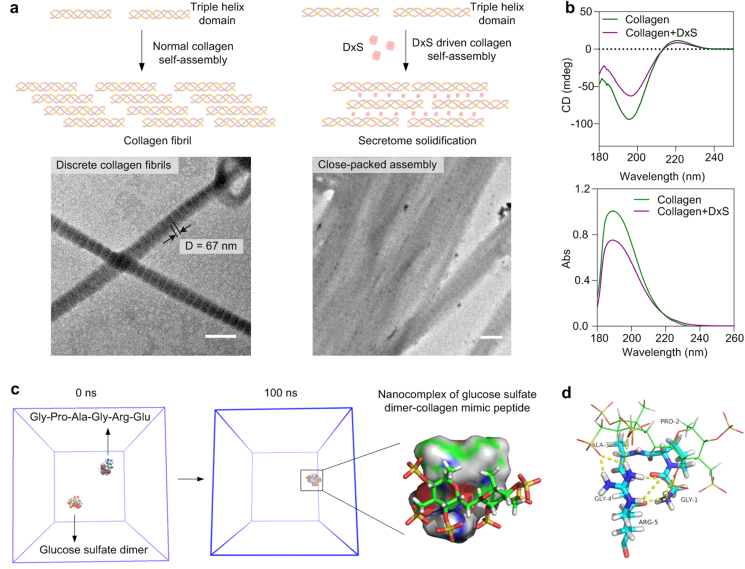
Co-assembly of collagen and dextran sulfate and molecular motifs. (a) Transmission electron microscopy imaging of the effect of dextran sulfate (DxS, 10 μg/ml) on collagen type I fibrillogenesis, visualized by negative staining with phosphotungstic acid (Top row: graphic depiction of potential co-assembly process). Scale bar = 250 nm. (b) Analysis of the effect of dextran sulfate on the molecular assembly of collagen type I (0.2 mg/ml) based on far-UV and circular dichroism (CD). (c and d) Simulation of the molecular dynamics of dextran sulfate collagen type I interaction using a glucose sulfate dimer and a typical collagen type I hexapeptide Gly-Pro-Ala-Gly-Arg-Glu, respectively.

Collagen type I, a major secreted ECM component of MSCs, was utilized as a model substrate to investigate the interaction with dextran sulfate in more detail and analyzed at an ultrastructural level. To this end, fibrillogenesis of purified collagen type I at physiological pH (pH 7.4) and 37 °C in the absence and presence of dextran sulfate was examined by phosphotungstic acid negative staining and transmission electron microscopy. In the absence of dextran sulfate, discrete fibrils of triple helical collagen type I were seen, exhibiting the expected cross-striation pattern with a band-width of 67 nm [51]. In the presence of dextran sulfate, collagen fibrils with a comparable diameter formed, also displaying typical cross-striations, although the pattern appeared less defined (Fig. 2a). Dextran sulfate was shown to interact [28] and co-deposit with collagen type I (Fig. 1h), suggesting that dextran sulfate may partially intercalate between collagen type I triple helices, resulting in reduced cross-striations during fibril formation (as illustrated in the graphic depiction Fig. 2a). The resulting collagen type I fibrils were further observed to assemble into tightly packed structures in the presence of dextran sulfate (Fig. 1, Fig. 2a), suggesting that dextran sulfate promoted the aggregation of formed collagen fibrils. Similar observations were previously made for heparin [52].

Circular dichroism (CD) spectroscopy was next employed to investigate the conformational change of collagen type I in the presence of dextran sulfate. After supplementation of dextran sulfate, collagen type I exhibited an absorption peak at 180–230 nm in the UV spectrum and two characteristic peaks at 196 and 221 nm in the CD spectrum, showing minimal change when compared with native collagen type I (Fig. 2b). These results suggested that the interactions between dextran sulfate and collagen type I did not alter its backbone structure [53]. The increase in R_pn_ (from 0.12 to 0.14) suggested an aggregation of collagen molecules [54], which was consistent with the ultrastructural observations (Fig. 2a).

To get detailed insights into the intermolecular interactions of collagen type I and dextran sulfate during the particle formation, molecular dynamics (MD) simulation was carried out using a simplified model constructed with a collagen-mimicking hexapeptide Gly-Pro-Ala-Gly-Arg-Glu [55] and a glucose sulfate dimer. A glucose sulfate dimer was used as a minimal molecular model of dextran sulfate. MD simulation showed that the glucose sulfate dimer formed intermolecular interactions with the hexapeptide spontaneously within 30 ns (Fig. 2c). The glucose sulfate dimer could spontaneously co-assemble with Gly-Pro-Ala-Gly-Arg-Glu through multiple interactions including van der Waals force, hydrogen bonding, and electrostatic interactions. Nonetheless, hydrogen bonding between the oxygen atom of the sulfate group and the hydrogen in Gly and Arg was the dominant interaction. Furthermore, the glucose sulfate dimer could also participate in the intermolecular interactions among the residues of Gly, Pro, Ala, and Arg (Fig. 2d). The interaction energy of the glucose sulfate dimer and the peptide was calculated as −59.808 kJ/mol. Of note, the investigated amino acid sequence was also suggested to act as a binding site for heparan sulfate [56].

### 3.3 Characterization and bioactive properties of MIPSOS

**Fig. 3:**
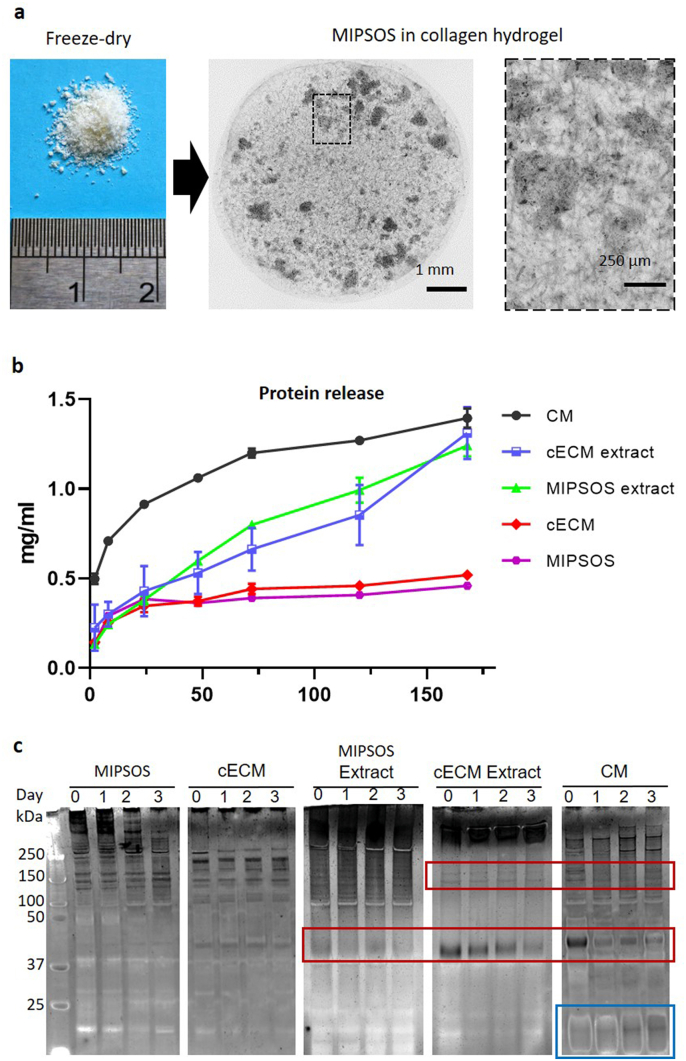
Stability of ECM-based microparticles. (a) Hydrogel construct with MIPSOS. Lyophilized cECM and MIPSOS resembled insoluble microparticles and could be encapsulated in a collagen type I scaffold. (b) Protein release profiles. Protein content released from cECM and MIPSOS entrapped in collagen hydrogels into the supernatant was estimated using BCA assay. (c) Protein degradation profiles. The microparticle formulation of MIPSOS and cECM protected protein contents from degradation, as compared to corresponding urea-solubilized extracts and MSC-conditioned medium (CM), when incubated at 37 °C for up to 3 days in PBS. Red and blue squares: Bands showing a marked decrease and increase, respectively, in density over time.

Assembled matrices deposited in the absence or presence of dextran sulfate were mechanically disrupted by being scraped into deionized water, collected and processed by lyophilization. This process resulted in the formation of cECM and MIPSOS microparticles, respectively. Both microparticle preparations exhibited a heterogeneous size distribution from sub-micrometer to sub-millimeter range (Fig. 3a). On average, 100 cm^2^ of MSC culture area yielded 1.65±0.27 mg of cECM, whereas dextran sulfate supplementation gave an increased yield of 2.12±0.39 mg with MIPSOS. The microparticles were insoluble in aqueous solution and therefore could be physically entrapped in a collagen type I hydrogel (Fig. 3a). For this, microparticles were mixed into a neutralized cold collagen I solution, which was then allowed to polymerize at 37 °C. Protein release profiles from MIPSOS and cECM, embedded in collagen hydrogels, demonstrated slow release kinetics into the supernatant. In contrast, conditioned medium supplemented into collagen hydrogels showed a burst release profile (Fig. 3b), while the release of cECM and MIPSOS urea-extract was steady but rapid. Likewise, no evident degradation of the solidified format of MIPSOS and cECM was observed, when incubated over several days in PBS at 37 °C (Fig. 3c). In contrast, continuous protein degradation was seen in MSC-conditioned medium as well as in urea-solubilized fractions of MIPSOS and cECM, evidenced as decreased density of higher molecular weight protein bands (Fig. 3c, red squares), with concurrent appearance of thicker lower molecular weight proteins bands, especially in conditioned medium (Fig. 3c, blue square).

**Fig. 4:**
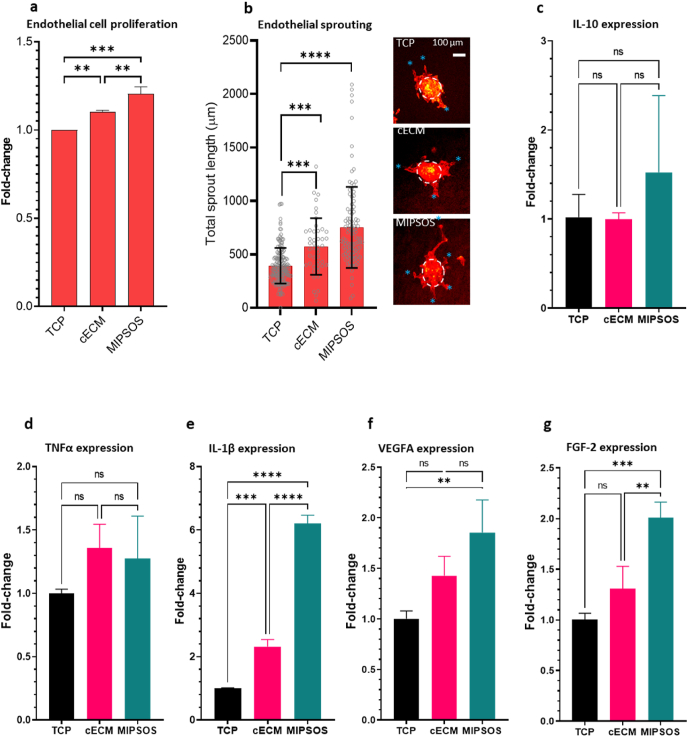
Bioactivity of ECM-based microparticles. (a) Endothelial cell proliferation on various substrates. HUVECs were seeded on TCP (control), cECM or MIPSOS and cultured for 3 days. Relative cell numbers were determined by CCK8 assay. (**b**) Endothelial sprouting assay on various substrates. The assay and quantification of cumulative sprout length/spheroid was performed using endothelial spheroids in a collagen hydrogel construct cast on tissue culture polystyrene (TCP), cECM or MIPSOS. Asterisks indicate tips of the formed sprouts. Scale bar = 100 μm. PCR for (c) IL-10, (d) TNFα, (e) IL-1β, (f) VEGFA and (g) FGF-2 expressed by THP-1 derived macrophages seeded on various substrates for 144 h.**, p < 0.01, ***, p < 0.001; ****, p < 0.0001.

Endothelial cells seeded on various substrates for 2 days showed an increased proliferation rate on cECM as compared to tissue culture polystyrene (TCP), which was further surpassed by cells cultured on MIPSOS (Fig. 4a). Similarly, when endothelial spheroids, embedded in a collagen gel, were layered over different types of microparticles, MIPSOS significantly promoted endothelial cell sprouting *in vitro*. This was evident in the cumulative sprout length per endothelial spheroid for MIPSOS, which exceeded that observed in collagen gels layered on TCP, and even over cECM (Fig. 4b).

Macrophages are one of the major cell types that interact with and degrade implanted materials, while directing wound processes via the signals they release [57]. Hence, THP-1-derived macrophages were seeded on the various substrates and their gene expression for pro- and anti-inflammatory as well as pro-angiogenic factors were investigated (Fig. 4c–g). No differences in the gene expression of IL-10 and TNF-α was observed, while an upregulation of pro-inflammatory and pro-angiogenic IL-1β was found in macrophages cultured on cECM and further exceeded on MIPSOS (Fig. 4c–e). Furthermore, alleviated mRNA levels of potent pro-angiogenic factors VEGFA and FGF-2 were found in macrophages cultured on MIPSOS (Fig. 4f and g).

### 3.4 MIPSOS promoted revascularization and healing *in vivo*

**Fig. 5:**
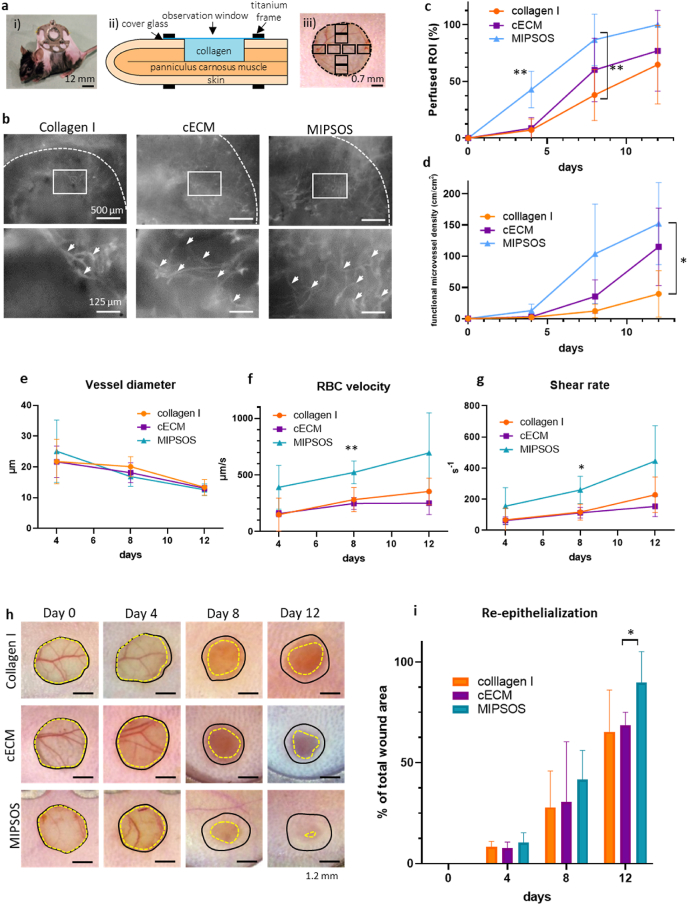
**MIPSOS promoted revascularization *in vivo*.** (**a**) Representative illustration of the experimental *in vivo* model, showing (i) a C57BL/6 mouse with dorsal skinfold chamber, (ii) cross-sectional schematic of skinfold chamber set-up and (iii) a full-thickness skin defect with indicated regions of interest (ROIs, black squares), which were analyzed by means of intravital fluorescence microscopy. (**b**) Intravital fluorescence microscopy of ingrowing perfused micro-vessels after intravenous injection of 5% FITC-labeled dextran 150,000 in full-thickness skin defects (Ø 3 mm) on day 8 after implantation. Dashed lines indicate the initial defect margins. White arrow heads point to vessel ramifications. (**c**) Percentage of perfused ROIs and (**d**) functional microvessel density (in cm/cm^2^) in wounds. (**e**) Diameter (μm) and (**f**) centerline red blood cell (RBC) velocity (μm/s) of 40 randomly selected microvessels. (g) Wall shear rate (y, in s^−1^) calculated from values obtained in (e) and (f) by means of the Newtonian definition y = 8 × v/d. (**h**) Wound re-epithelialization of full-thickness skin defects over a period of 12 days. Black lines indicate initial defect margins. Yellow dashed lines indicate progression of re-epithelialization. (**i**) Quantification of wound area over time * p < 0.05; **p < 0.01.

The *in vitro* functional angiogenesis assay data prompted us to further investigate the pro-angiogenic potential of MIPSOS *in vivo* using a murine dorsal skinfold chamber model (Fig. 5a (i)). This model is based on creating full-thickness skin wounds fixed within a dorsal frame (Fig. 5a (ii)). The frame inhibits wound healing by contraction, while enabling real-time intravital imaging of discrete regions of interest (ROIs) in the healing wound (Fig. 5a (iii)) [58,59]. The created full-thickness skin wounds were filled with either empty collagen type I hydrogel (1.8 mg/ml), or collagen type I hydrogels loaded with cECM or MIPSOS at a concentration of 10 mg/ml (Fig. 5a (ii)). Collagen I was used as a delivery vehicle, as it is known for its low antigenicity and inherent biocompatibility and has been widely utilized to deliver cells and other bioactive factors in research as well as a commercial setting [60]. Moreover, pure collagen-based scaffolds have been shown to not significantly promote revascularization and skin wound healing [61], rendering it an advantageous delivery vehicle in our set-up. For continuous real-time assessment of the revascularization, mice were injected intravenously with fluorescently labeled dextran to visualize perfused and, thus, functional blood vessels. Eight standardized ROIs (Fig. 5a (iii)) were repeatedly imaged at different time points by intravital fluorescence microscopy. All wounds were gradually revascularized by microvessels growing from the wound edges towards the center. In comparison to empty hydrogels and cECM-treated wounds, MIPSOS treatment showed the most advanced functional capillary network with an increased number of ramifications (Fig. 5b, white arrows). Indeed, MIPSOS treatment significantly increased the percentage of perfused ROIs throughout the study, and only wounds treated with MIPSOS exhibited 100% revascularization by day 12 (Fig. 5c). A similar, quantifiable trend was observed with functional microvessel density, which was increased in cECM-treated wounds on day 8 and 12, but surpassed by MIPSOS treatment at all time-points (Fig. 5b,d). Although the diameters of individual microvessels were comparable in all treatment groups (Fig. 5e), MIPSOS-treated wounds consistently showed higher perfusion, as evidenced by an increased average RBC velocity (Fig. 5f) and resultant wall shear rates (Fig. 5g). In agreement with the enhanced revascularization observed, wounds treated with MIPSOS exhibited the fastest re-epithelialization with a 90% closure by day 12, significantly exceeding re-epithelialized areas in cECM-treated wounds. In contrast, cECM treatment did not differ from empty collagen type I hydrogel-treated controls (Fig. 5h and i).

**Fig. 6:**
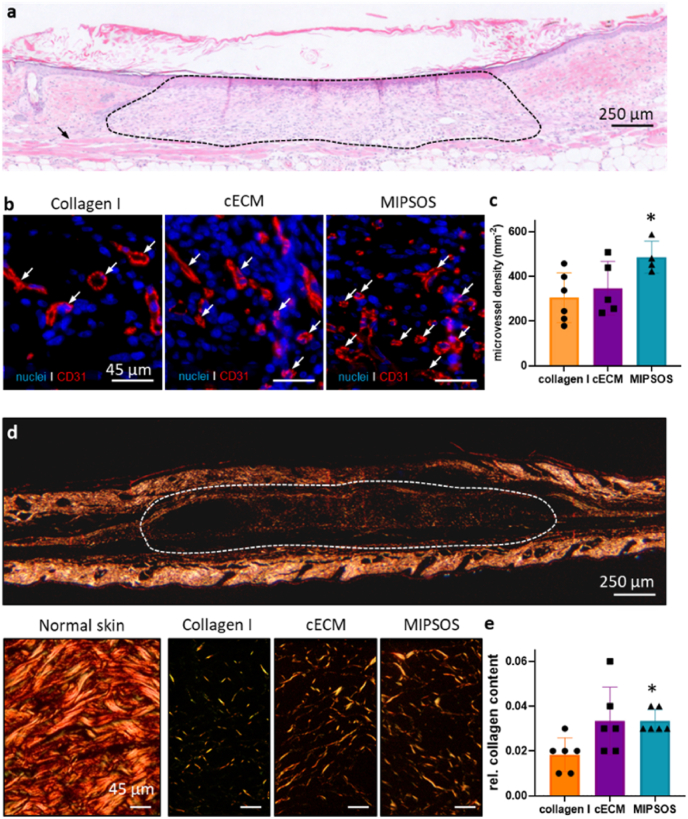
MIPSOS promoted wound healing. (a) Representative HE-stained histological sections of full-thickness mouse skin wounds treated with MIPSOS particle-laden collagen gel on day 12. The area of hydrogel implant is indicated by dashed line. Bar = 250 μm. (b) Representative images of CD31-stained microvessels in skin defects on day 12; nuclei were counterstained with DAPI. Bar = 45 μm. (c) Quantified microvessel density in mm^−2^. (d) Polarized light microscopy of Sirius red-stained sections of normal skin as well as healed skin defects on day 12. Bar = 250 μm (top) or 45 μm (bottom). (e) Corresponding quantification of relative collagen signal (implant/skin) on day 12 after implantation. *, p < 0.05, compared to collagen I.

Histology of wound areas showed intense cellular infiltration on day 12. However, no visual differences were observed for the different conditions (Fig. 6a, suppl. Fig. S2). Semi-quantitative analysis of CD31-stained microvessel density (Fig. 6b) demonstrated significantly more blood vessels per area in MIPSOS-treated wounds as compared to empty hydrogel. Wounds treated with cECM exhibited a trend towards a higher microvessel density; however, these results were not statistically significant (Fig. 6b and c). These histological findings are in good agreement with the results from the perfusion studies in live animals using intravital fluorescence microscopy (Fig. 5b). Sirius red staining was utilized to visualize mature collagen fibers under polarized light to assess the maturation state of the granulation tissue (Suppl. Fig. S3). Measured collagen content in wound areas was normalized to the collagen content of the adjacent healthy tissue. Image analysis of representative ROIs (Fig. 6d and e) as well as a semi-quantitative analysis of collagen content revealed that wounds treated with MIPSOS-laden hydrogels led to significantly more mature collagen fiber deposition as compared to vehicle control (empty collagen hydrogel) (Fig. 6e). These data suggest that MIPSOS stimulated the synthesis of *de novo* granulation tissue.

### 3.5 Enriched pro-angiogenic extracellular proteins in MIPSOS

The intrinsic pro-angiogenic properties of cECM and MIPSOS are plausibly linked to the active components of the solidified MSC secretome and their subsequent release. However, the substantially stronger pro-angiogenic bioactivity of MIPSOS suggests a role of dextran sulfate in the enrichment of pro-angiogenic components. To test this hypothesis, label-free quantitative proteomic analysis of MIPSOS and cECM was performed. As the insoluble nature of the ECM renders proteomic analysis challenging, equal amounts of cECM and MIPSOS were partially solubilized with urea. Furthermore, the soluble fraction was optionally fractionated and the unfractionated and the fractionated soluble fraction, as well as the insoluble fraction underwent enzymatic digestion and proteomic analysis independently. As three independent samples for each condition were analyzed up to 9 data points for each protein were derived.

**Fig. 7:**
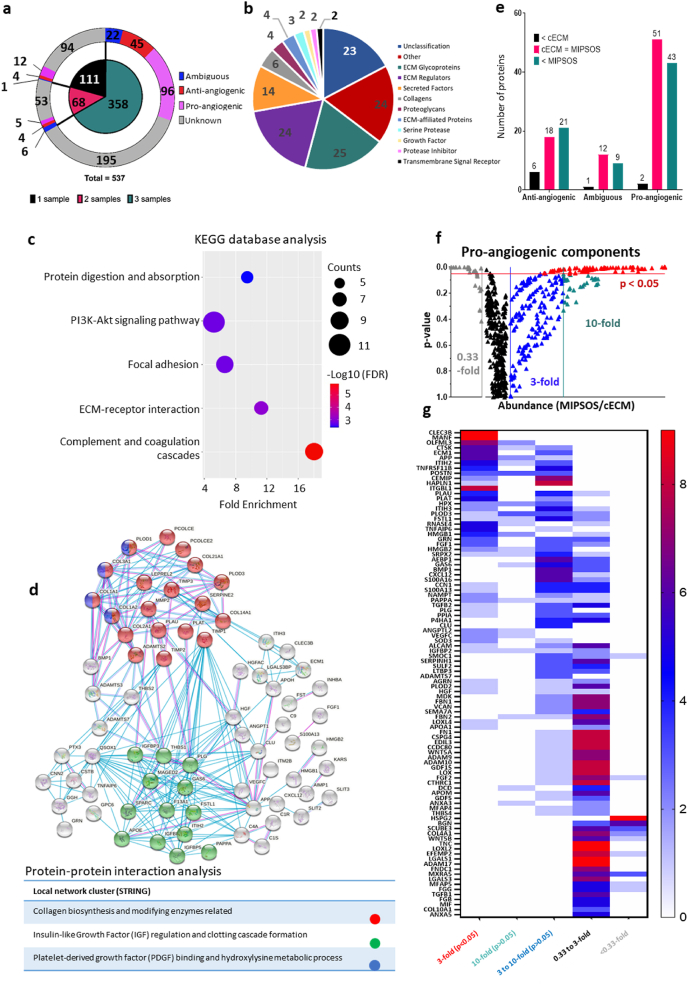
Pro-angiogenic components are enriched in MIPSOS. (a) Pie diagram visualizing distribution of identified secretome proteins from three biological replicates according to their reported role in angiogenesis. (b) A total of 133 proteins with MIPSOS/cECM abundance ratio >3 folds were further classified based on Matrisome Project and Panther classification system. (c) Using KEGG database in DAVID bioinformatics, 5 pathways (FDR <0.05) were identified and organized by false discovery rate FDR value as well as protein counts. (d) Protein-protein interaction (PPI) based on STRING Network analysis (Confidence score threshold at 0.7 (high)) highlights significant protein interaction networks. Proteins are represented as nodes of different colors. (e) Number of proteins identified in all three biological replicates (from a) categorized based on their enrichment (3-fold difference) in cECM (<cECM), MIPSOS (<MIPSOS) or comparable abundance in both (cECM = MIPSOS), as well as reported role in angiogenesis. (f) Volcano plots of protein abundance (MIPSOS/cECM) against their respective p-value. Arbitrary thresholds to determine distinct levels of confidence are set as 0.33 (equivalent to 3-fold abundance in cECM), 3-fold, 10-fold and p < 0.05. Each protein is represented by up to 9 data points (3 biological replicates x 3 types of sample processing (urea-insoluble, urea-soluble, urea-soluble/fractionated)). (g) Heat map depicting number of data points of pro-angiogenic proteins present in the groups with distinct levels of confidence.

Analysis of three biologically independent replicates identified 537 secreted human protein components, with 358 of these being commonly picked up in all three biologically independent samples (Fig. 7a). Of these, 133 were enriched in MIPSOS ≥3-fold over cECM, and could be identified as core matrisome or -associated factors, including collagens, glycoproteins, proteoglycans and growth factors (Fig. 7b). Characterization of these selected proteins based on gene ontology (GO) analysis further confirmed them as cellular secretome components, particularly of the ECM. Interestingly, many of the enriched proteins were indeed sulfate-, heparin- and/or glycosaminoglycan-binding, suggesting possible capture via direct interaction with dextran sulfate (Suppl. Fig. S4).

Pathway analysis identified a group of signaling proteins enriched in MIPSOS (mainly growth factors), such as angiopoietin 1, FGF1, HGF and VEGF C. These are implicated in the PI3K-Akt pathway (Fig. 7c), known to promote cell migration, proliferation and angiogenesis - key biological processes during wound healing [[62], [63], [64], [65]]. Other enriched proteins were implicated in focal adhesions and ECM-receptor interactions, potentially facilitating ECM-cell interactions (Fig. 7c). Furthermore, MIPSOS were found to be enriched in proteins involved in the complement and coagulation cascade (*i.e*., various complement factors and plasminogen activators) - important signals for wound healing and angiogenesis [66,67] (Fig. 6c). While components of the complement and coagulation cascades were previously identified in the MSC transcriptome [68,69], they are also found in serum. As ECM-based microparticles were assembled at low serum concentrations (0.5%), we cannot completely rule out that some of these components might have been carried over from fetal bovine serum and falsely identified as of human origin due to close interspecies homology.

Protein-protein interaction analysis with a confidence ratio of 0.7 highlighted that enriched proteins had strong interactions with protein clusters such as collagen assembly and growth factor (e.g., IGF and PDGF) regulations (Fig. 7d).

Next, we focused on protein components that had been already implicated in angiogenesis. Abundance comparison of components with an ambiguous or antagonistic role in angiogenesis indicated them to be slightly more abundant in MIPSOS as compared to cECM. In contrast, MIPSOS contained 20-times more pro-angiogenic proteins than cECM and thus in general a higher variety of secreted proteins (Fig. 7e). To identify the most strongly enriched pro-angiogenic components among them, we applied an additional, higher level of confidence at 10-fold abundance in MIPSOS over cECM. This protein selection contained the majority of proteins, which were significantly (p < 0.05) more abundant in MIPSOS as compared to cECM (Fig. 7f). The resultant selection of enriched pro-angiogenic components comprised 46 proteins (Fig. 7g), that may be assumed to be the basis for the superior pro-angiogenic activity of MIPSOS. Here, FGF-1, HGF, angiopoietin 2 (ANGPT2) [21,22] and periostin (POSTN) [70], all pro-angiogenic components previously reported for MSCs stood out (Fig. 7g). Other functional molecules, previously associated with MSCs, were vastly enriched in MIPSOS, including TSG-6 [21,22], progranulin (GRN) [71], CCN1 [72] and OLFM3 (OLFML3) [73]. In addition, many pro-angiogenic factors such as MANF [74], ECM1 [75]. HMGB1 and HMGB2 [76] and GAS6 [77], hitherto not associated with MSCs, were found to be enriched in MIPSOS, suggesting novel players potentially responsible for the pro-angiogenic potential of MSCs (Fig. 7g, Suppl. Fig. 5, Fig. 6).

## 4 Discussion

In an attempt to address the major limitations of experimental approaches to restore or induce vascularization, we leveraged the dextran sulfate-mediated supramolecular co-assembly of microparticles comprising MSC-derived ECM, thereby creating a solidified secretome. Dextran sulfate's ability to mimic heparan sulfate facilitated the enrichment pro-angiogenic components into microparticles, augmenting their efficacy to promote revascularization and wound healing.

The MSC-derived secretome, beneficial for regeneration and repair, stimulated considerations of potential benefit of local implantation of MSCs into ischemic tissue. Later, in an effort to address the low cell engraftment rates and streamline regulatory and off-the-shelf approaches, harvesting the MSC secretome for local application was explored [78,79]. Unfortunately, the efficacy of MSC-conditioned media [78,79] or isolated components thereof, was hampered by the rapid clearance of components [80]. Attempts to stabilize MSC-derived soluble factors utilized carriers such as hydrogels [81] or particles [82]. Unfortunately, those approaches generally did not exhibit the necessary complexity to sequester, present and modulate the bioactivity of the complex cellular secretome. Furthermore, these approaches only considered the immediately soluble fraction of the MSC-derived secretome.

MSCs are excellent synthesizers of ECM, which represents the insoluble fraction of the cellular secretome. Insoluble and soluble secretome fractions share components, but the ECM accumulates additional bioactive components, while being properly assembled into a three-dimensional network and thus solidified [83]. As demonstrated here, this insoluble format greatly protected proteins from degradation and enabled their sustained release, which was shown to be crucial for achieving long-term therapeutic effects [4,5,7]. Moreover, cell-derived ECM and MIPSOS arising therefrom should at least partially recapitulate the complex biological machinery and thus complex signaling of native tissue ECM [25].

Cell-derived ECM (mainly from fibroblasts and MSCs) has been previously shown to promote cellular proliferation and migration of major cell types involved in skin wound healing such as fibroblasts and endothelial cells, as well as exhibit a pro-angiogenic potential *in vitro* [[84], [85], [86], [87]]. Initial studies further confirmed a pro-angiogenic potential *in vivo* in a chick chorioallantoic membrane (CAM) assay [26] or when co-transplanted with pre-vascularized collagen constructs into skin wounds [87]. When applied to skin wound healing fibroblast-as well as MSC-derived ECM showed a trend towards better wound healing, although no significant differences in wound closure or revascularization were evident as compared to delivery vehicles lacking the respective cell-derived matrices [84,88]. This is in accordance with the results we have obtained with the unmodified cECM, clearly indicating that further enhancement/programming of matrix bioactivity is necessary for successful skin wound revascularization and healing.

The insoluble microparticle formulation reported here favorably enabled and enhanced the desired cell-responsive release of bioactive factors by bioresorption. This is of particular interest, as an ischemic injury is accompanied by an elevated inflammatory response, the intensity of which is proportional to the level of ECM degrading enzymes [89]. Hence, a more severe ischemic injury would result in a more rapid degradation of microparticles, thus quickening the release of a large repertoire of bioactive factors.

Using collagen type I as a representative major structural ECM component, we investigated the role of dextran sulfate in collagen fibrillogenesis. Indeed, dextran sulfate did not inhibit collagen type I fibrillogenesis during their co-aggregation. Furthermore, it converged and facilitated physical interactions between collagen fibrils, promoting the formation of structured assemblies. The resulting typical granular structures were also observed in cell cultures, suggesting a comparable mechanism of co-deposition. This is in accordance with previous reports, where negatively charged polysaccharides were even suggested to accelerate collagen fibril formation [90].

Dextran sulfate has been previously shown to promote ECM deposition *in vitro*, albeit it was attributed to a macromolecular crowding effect [39,[47], [48], [49]]. Besides dextran sulfate, other negatively charged polyelectrolytes have been suggested to have a similar effect. These include large seaweed-derived sulfated polysaccharides such as carrageenan [91,92], fucoidan [93], galactofucan and ulvam, as well as fucan [94]. ECM-derived hyaluronic acid was also shown to enhance ECM deposition *in vitro* [95,96], although its intrinsic bioactivity suggested various possible mechanisms including its direct involvement in fibronectin fibrillogenesis [97]. For other smaller ECM-derived glycosaminoglycans such as heparin, chondroitin sulfate and dermatan sulfate varying results were obtained, including that, while collagen I deposition was inhibited, the deposition of a 60 kDa collagen species was enhanced [98]. Since dextran sulfate was shown to co-assemble with secreted ECM components [28,50], especially the larger negatively charged macromolecules mentioned above were likely facilitating ECM deposition in a comparable manner, resulting in biomaterials with similar bioactivity. Since dextran sulfate is a biodegradable, highly branched and sulfated glucan and its building blocks (glucose molecules) thus natural and metabolizable components of the cell, it exhibits very good biocompatibility. It is thus one of the most advantageous sulfated polysaccharides for implantation as part of a biomaterial or biologic.

We have shown here and earlier [28] that dextran sulfate is incorporated into matrix assemblies and thus sought to take advantage of its ability to bind pro-angiogenic factors. It is noteworthy that dextran sulfate also promoted the signaling activity of these factors and prolonged their half-life, suggesting that dextran sulfate can act as a heparan sulfate mimetic [[29], [30], [31], [32], [33], [34]]. Indeed, dextran sulfate was also previously utilized in polyelectrolyte complexes to retain growth factors [[99], [100], [101], [102]]. We showed here that the co-assembly of intrinsically pro-angiogenic MSC-derived ECM with dextran sulfate to form MIPSOS resulted in a superior pro-angiogenic efficacy *in vitro* and *in vivo*.

*In vitro* assays further revealed that MIPSOS did not only directly affect endothelial cells, but also macrophages, which upon interaction with MIPSOS increased their expression of pro-angiogenic factors such as VEGF, FGF-2 and IL-1β. Although IL-1β is pro-inflammatory, other pro-inflammatory factors such as TNFα were not upregulated, suggesting that macrophages were not polarized in classically activated M1 macrophages. Since macrophages are one of the major cell types that interact and degrade implanted materials, while modulating the progression of the wound healing process via their secretome [57], current data suggest that MIPSOS might simultaneously promote angiogenesis by directly affecting endothelial cells and indirectly via macrophages.

Revascularization is a multi-step process guided by a balance of pro-angiogenic and anti-angiogenic factors, both found in cECM and MIPSOS. It is intriguing that proteomic analysis revealed an accumulation of a higher variety of pro-angiogenic than anti-angiogenic factors. This may reflect the overall pro-angiogenic secretome of MSCs [21,22]. It is thus plausible that the superior pro-angiogenic efficacy of MIPSOS is derived from the huge portfolio of enriched bioactive components, which are not only captured in a sustainable release form, but also present in appropriate stoichiometric proportions to each other. It should be noted that the superior ability of MIPSOS to promote revascularization over cECM is likely a result of the synergistic interplay of a large variety of bioactive components, rather than few selected ones. This intrinsic complexity also renders MIPSOS superior to other compositionally more limited approaches (mono- or oligo-factor administration), which in contrast have to be administered at supraphysiological doses to exhibit any effect.

Nonetheless, the intrinsic complexity and insolubility of cell-derived ECM renders proteomic analysis to be challenging. Especially bioactive, non-structural ECM components, which are naturally only present in low amounts within the ECM can be missed in this approach. As only factors identified in all three biologically independent samples were considered, the presented list of factors is finite. Some of the protein components identified here have already been associated with the MSC-mediated revascularization promoting mechanism of action. Nonetheless, many additional pro-angiogenic components previously not linked with MSCs, as well as ECM components with unknown role in angiogenesis represent potential new factors responsible for the superior pro-angiogenic properties of MIPSOS. In future studies the accumulation of pro-angiogenic factors may be further enhanced by prior activation of MSCs. It is worth to mention that since dextran sulfate is retained within the deposited ECM might, it may also exhibit some direct bioactivity, as described earlier [103]. Nonetheless, even assuming that all supplemented dextran sulfate was incorporated into the microparticles during assembly, none was degraded, engulfed and digested during cell culture or lost during decellularization, maximally 8 μg dextran sulfate, with the actual amount being a fraction of this, would have been supplemented into the wounds. This is many-fold less than what was needed to be repeatedly used to treat wounds in rats [103]. As dextran sulfate was reported to exhibit good biocompatibility and to be used as a plasma volume expander and anticoagulant [104], we did not expect nor observe any adverse effects from the potentially co-transplanted dextran sulfate. Since dextran sulfate is susceptible to hydrolysis and cellular uptake with subsequent lysosomal degradation [105], remaining dextran sulfate residues are expected to be cleared from tissue. Hence, it is currently assumed that the observed bioactivity and pro-healing effect is driven by the cell-derived protein components, which make up the majority of MIPSOS. Naturally, other non-protein components, such as ECM-bound nanovesicles [106], not investigated here, might also have contributed to the observed bioactivity.

## 5 Conclusion

We have established an approach to produce hierarchically nanostructured microparticles (named MIPSOS), based on the dextran sulfate-mediated co-assembly of cell-derived ECM. The interconnected and thus insoluble microstructure of MIPSOS protected the bioactive protein components from rapid degradation and enabled their sustained release. Simultaneously, dextran sulfate's ability to mimic heparan sulfate promoted capturing of a plethora of pro-angiogenic factors onto the ECM backbone, resulting in superior pro-angiogenic properties and therapeutic potential. We anticipate that this approach will open avenues for the synthesis of a new class of biomaterials based on solidified cell-derived secretome with tailored bioactivity, thus addressing a major limitation of stem cell-based therapy. The presented findings highlight the development of a ready-to-use particle-based platform, tailored to exhibit improved pro-angiogenic properties for a range of clinical challenges related to revascularization.

## Authors’ contribution

T.S. performed *in vivo* analysis. M.A. produced microparticles and performed *in vitro* characterization and analysis. G.G. performed molecular analysis of dextran sulfate and collagen I interaction and was supported by X.W. Y.Y.C. and K.-HK. performed proteomics analysis. K.K.L., Y.R. and M.D.W. analyzed proteomic data. Y.L. performed degradation and release studies. C.H.K.Y. optimized urea extraction protocol. M.W.L. and M.D.M. contributed to *in vivo* data analysis and interpretation. M.R., and R.S.T. contributed to data interpretation and edited the manuscript. A.B. conceived the idea of this project and designed the study. J.G. contributed to study design. T.S., J.G., and A.B. interpreted data and wrote the manuscript.

## CRediT authorship contribution statement

**Thomas Später:** Validation, Investigation, Formal analysis, Writing – original draft, Visualization. **Marisa Assunção:** Validation, Investigation, Formal analysis, Writing – original draft, Visualization. **Kwok Keung Lit:** Investigation. **Guidong Gong:** Investigation, Writing – review & editing. **Xiaoling Wang:** Investigation. **Yi-Yun Chen:** Investigation, Formal analysis. **Ying Rao:** Formal analysis. **Yucong Li:** Investigation. **Chi Him Kendrick Yiu:** Investigation. **Matthias W. Laschke:** Formal analysis, Writing – review & editing. **Michael D. Menger:** Development or design of methodology, creation of models, Writing – review & editing. **Dan Wang:** Formal analysis, Writing – review & editing. **Rocky S. Tuan:** Formal analysis, Writing – review & editing. **Kay-Hooi Khoo:** Formal analysis, Writing – review & editing. **Michael Raghunath:** Formal analysis, Writing – review & editing. **Junling Guo:** Conceptualization, Formal analysis, Visualization, Funding acquisition, Writing – review & editing. **Anna Blocki:** Conceptualization, Formal analysis, Investigation, Writing – original draft, Visualization, Supervision, Project administration, Funding acquisition.

## Declaration of competing interest

AB and MA would like to declare the filing of a PCT patent application (WO 2020/228733 A1).
