# Supplementary material for: Engineering microparticles based on solidified stem cell secretome with an augmented pro-angiogenic factor portfolio for therapeutic angiogenesis
Source: Bioact Mater. 2022 Apr 2;17:526–41. doi: 10.1016/j.bioactmat.2022.03.015 (PMC9270501; doi:10.1016/j.bioactmat.2022.03.015)
Supplement: Multimedia component 1 [file mmc1.docx]

Supporting Information


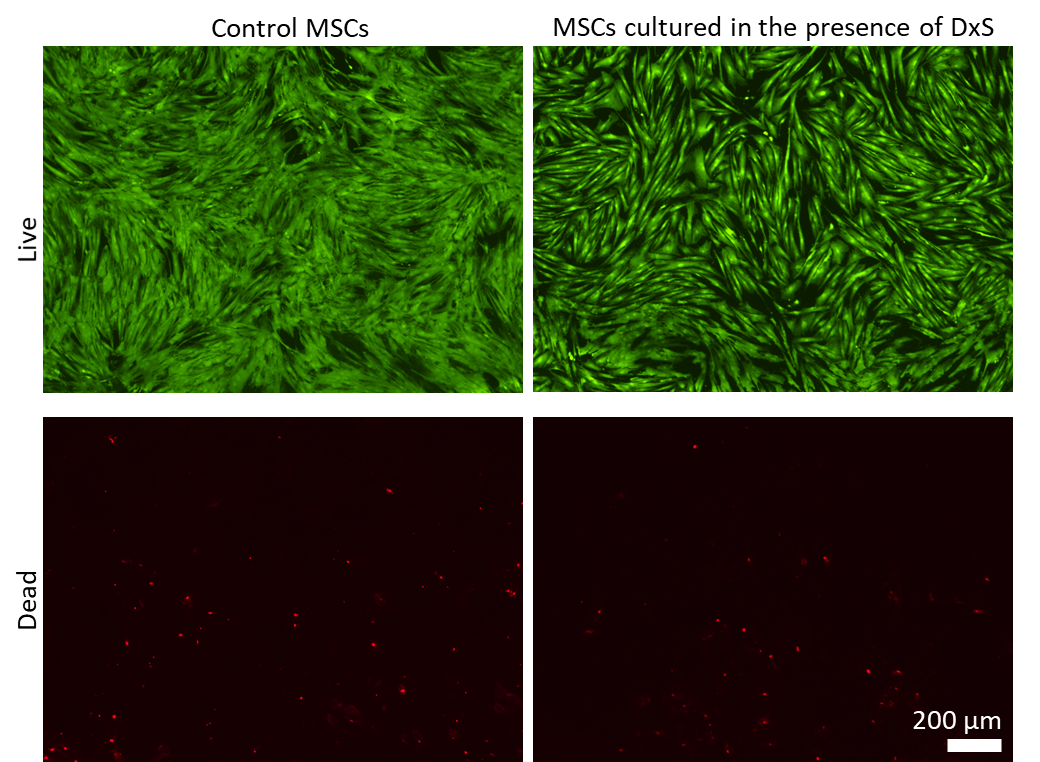


**Suppl. Figure S1:** MSCs exhibit high cell viability during ECM assembly in the presence and absence of dextran sulfate (DxS). MSCs (Passage 7) were cultured under low serum conditions (0.5% FBS) and under optional supplementation of dextran sulfate to deposit ECM. After 6 days of cultured MSCs were subjected to live/dead cell staining.


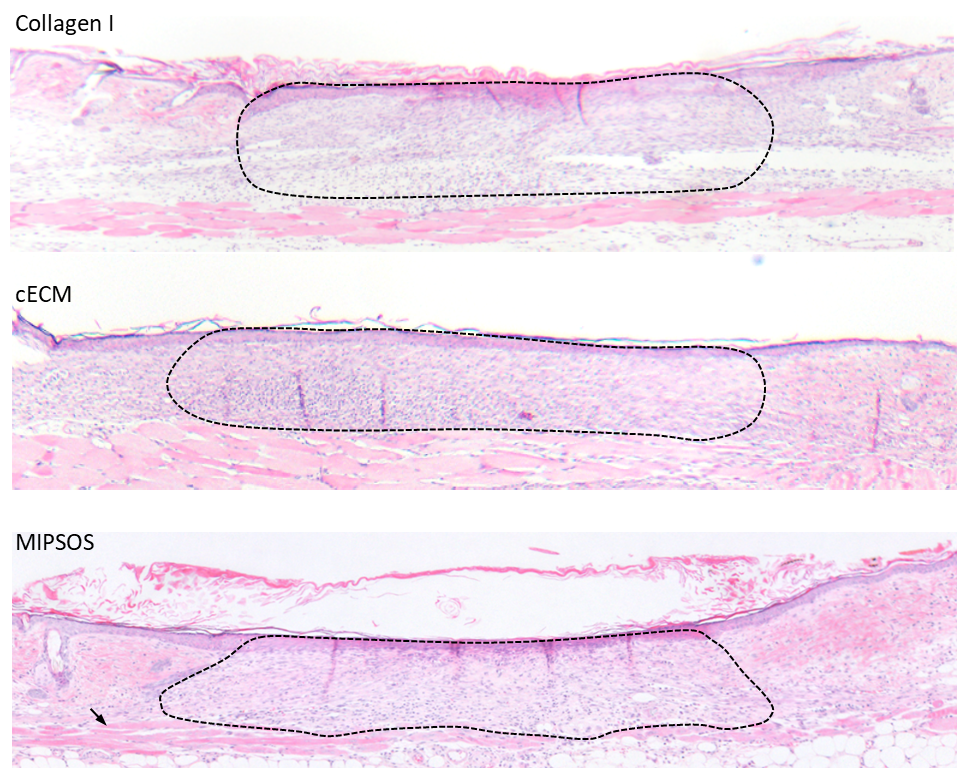


Suppl. Figure S2: Representative HE-stained histological sections of full-thickness mouse skin wounds treated with MIPSOS or cECM microparticle-laden collagen hydrogels on day 12. Empty collagen I hydrogels (Collagen I) were used as delivery vehicle controls. The area of hydrogel implant is indicated by dashed line. Bar = 250 µm.


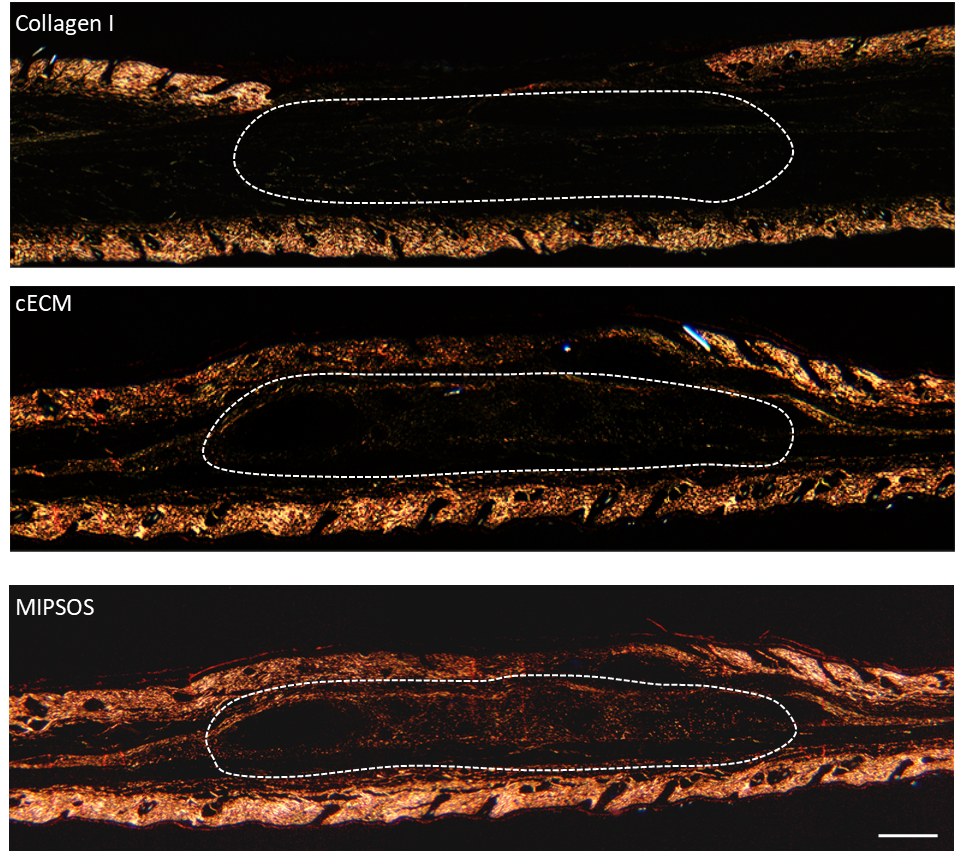


Suppl. Figure S3: Polarized light microscopy of Sirius red-stained sections of healed skin defects on day 12. The area of hydrogel implant is indicated by dashed line. Bar = 250 µm.


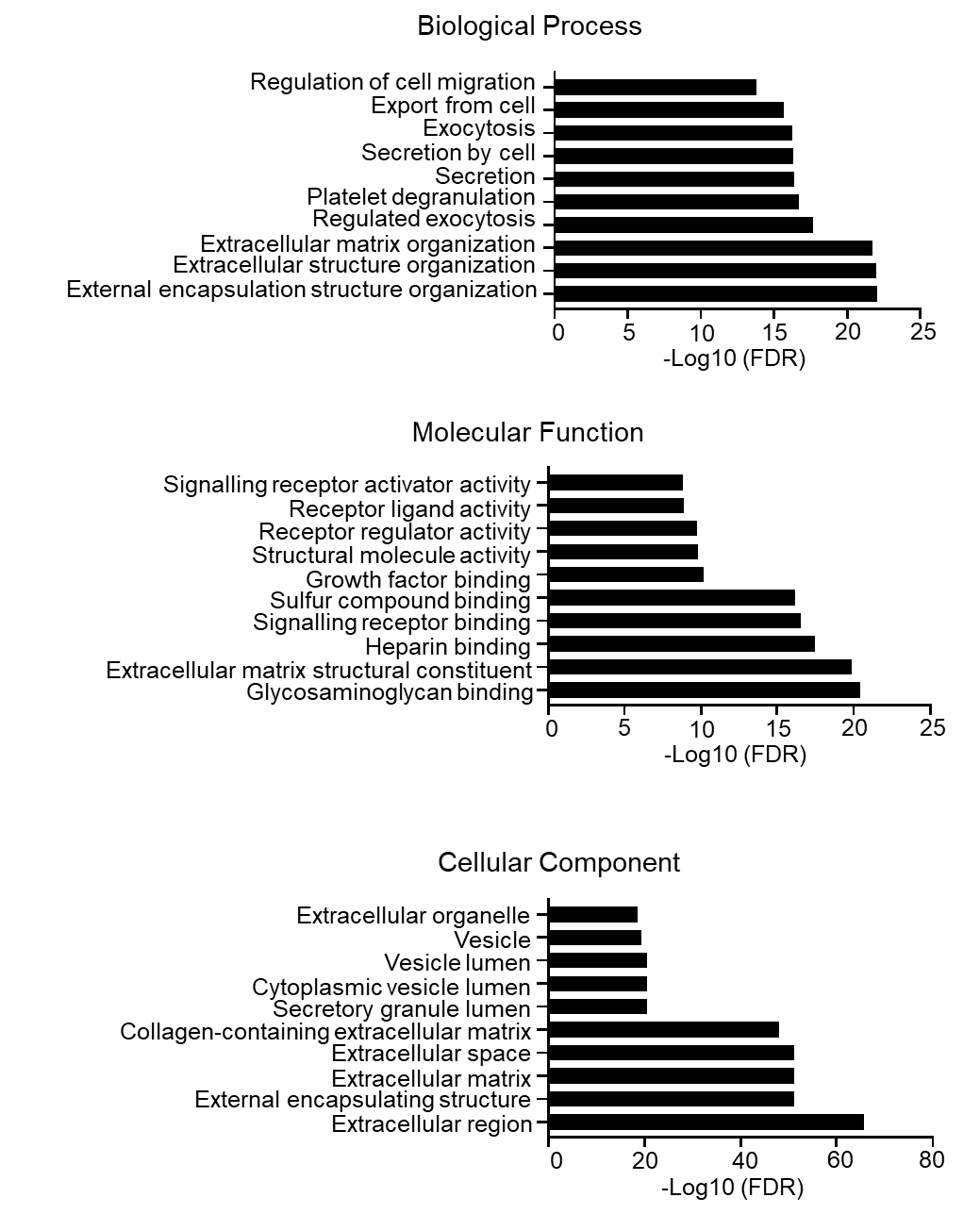


**Suppl. Figure S4:** Classification of differentially expressed proteins based on GO enrichment analysis. The 10 most significantly (FDR<0.05) enriched GO terms in biological process, molecular function and cellular component are presented. All the FDR of the terms was negative 10-base log transformed.


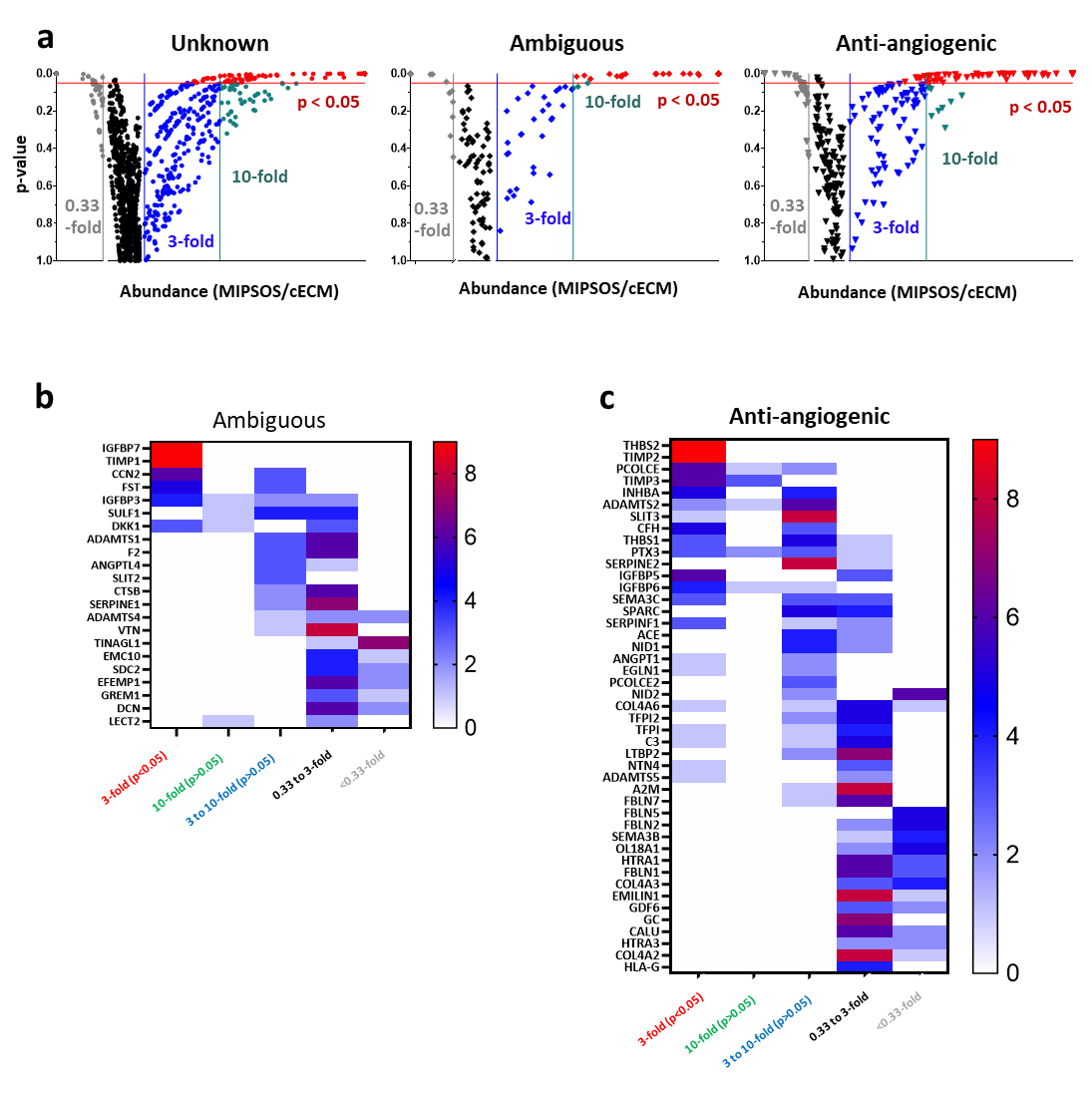


**Suppl. Figure S5:** MIPSOS exhibit a large variety of secreted components. a) Vulcanoplots plotting the protein abundance (MIPSOS/cECM) of components with a reported unknown (left), ambiguous (middle) and anti-angiogenic (right) role in angiogenesis against their respective p-value. Following arbitrary thresholds to determine distinct levels of confidence are set: 0.33 (equivalent to 3-fold abundance in cECM), 3-fold, 10-fold and p < 0.05. Each protein is represented by up to 9 data points (3 biological replicates x 3 types of sample processing (urea-insoluble, urea-soluble, urea-soluble/fractionated). b) and c) Heatmap depicting number of data points of proteins with a reported ambiguous and anti-angiogenic role present in the groups with distinct levels of confidence, respectively.


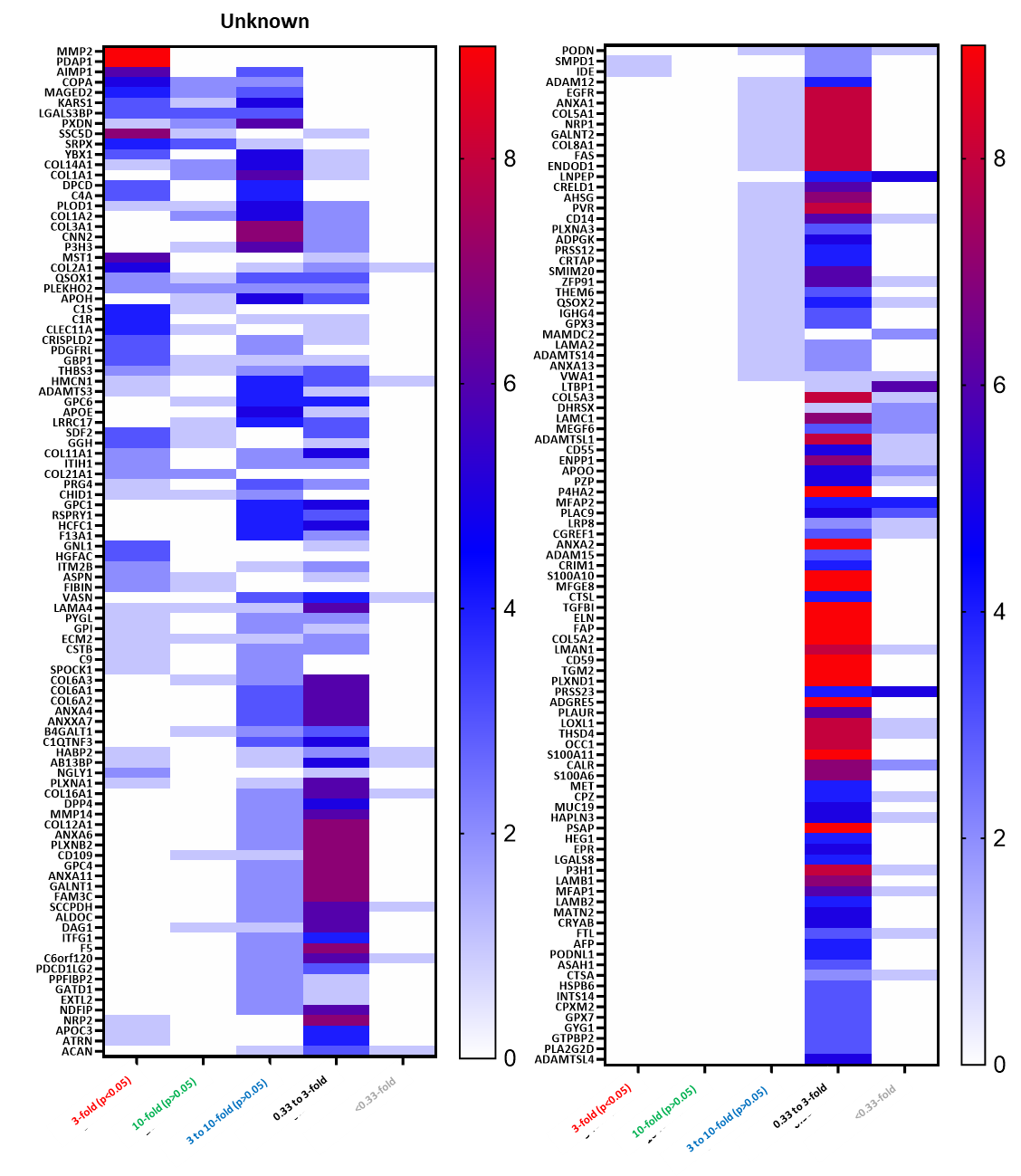


**Suppl. Figure S6:** MIPSOS contain a large amount of secreted proteins with unknown role in angiogenesis. Heatmap depicting number of data points of proteins with an unknown role in angiogenesis present in the various abundances in MIPSOS and cECM. Following arbitrary thresholds to determine distinct levels of confidence are set: 0.33 (equivalent to 3-fold abundance in cECM), 3-fold, 10-fold and p < 0.05. Each protein is represented by up to 9 data points (3 biological replicates x 3 types of sample processing (urea-insoluble, urea-soluble, urea-soluble/fractionated).
